# Leptin Selectively Regulates Nutrients Metabolism in Nile Tilapia Fed on High Carbohydrate or High Fat Diet

**DOI:** 10.3389/fendo.2018.00574

**Published:** 2018-09-27

**Authors:** Cai-Zhi Liu, An-Yuan He, Li-Jun Ning, Yuan Luo, Dong-Liang Li, Mei-Ling Zhang, Li-Qiao Chen, Zhen-Yu Du

**Affiliations:** ^1^Laboratory of Aquaculture Nutrition and Environmental Health, School of Life Sciences, East China Normal University, Shanghai, China; ^2^Division of Endocrinology, Metabolism and Lipid Research, Washington University School of Medicine, Saint Louis, MO, United States; ^3^College of Marine Sciences, South China Agricultural University, Guangzhou, China

**Keywords:** leptin resistance, DIO teleost, selective regulation, lipid metabolism, glucose metabolism

## Abstract

Leptin is known to inhibit appetite and promote energy metabolism in vertebrates. Leptin resistance (LR) commonly occurs in diet-induced obesity (DIO) in mammals. However, the roles of leptin in the energy homeostasis in DIO animals with LR remain unclear. Here we first verified the high expression of leptin in subcutaneous adipose tissue (SCAT) as in liver in Nile tilapia. Furthermore, we produced two types of DIO Nile tilapia by using a high-carbohydrate diet (HCD) or a high-fat diet (HFD), and confirmed the existence of LR in both models. Notably, we found that HCD-DIO fish retained leptin action in the activation of lipid metabolism and showed LR in glucose metabolism regulation, while this selective leptin action between lipid and glucose metabolism was reversed in HFD-DIO fish. Fasting the fish for 1 week completely recovered leptin actions in the regulation of lipid and glucose metabolism. Therefore, leptin may retain more of its activities in animals with LR than previously believed. Evolutionally, this selective regulation of leptin in nutrients metabolism could be an adaptive mechanism in animals to store surplus calories when different types of food are abundant.

## Introduction

The cytokine leptin was first identified in mouse adipose tissue by positional cloning in 1994 (1). Mammalian leptin is an adipostatic hormone which plays a key role in feeding control and energy homeostasis (2). In most cases of obesity, the hyperleptinemia neither reduces appetite nor reverses weight gain. Under such condition, the administration of exogenous leptin has no effect. This condition has been termed as “leptin resistance” (LR) (3). Although several mechanisms of LR have been proposed (4), recent data obtained using a leptin receptor antagonist demonstrated that hyperleptinemic diet-induced obesity (DIO) mice retained leptin action (5). Consequently, the term “leptin resistance” with more than 4,000 citations has recently been redefined (6, 7). However, the role of leptin in DIO is still unclear. Most studies on LR have focused on exploring the mechanisms to reducing obesity using leptin, but ignored leptin original function from the perspective of adaptive evolution (8). Given that teleosts are the first true vertebrates on earth, the physiological roles of teleostean leptin should be original and best represented (9). Therefore, understanding the role of teleostean leptin in metabolic regulation could help to reveal the evolution of energy homeostasis in vertebrates (10). Moreover, a fish model could help to explore the original features of LR.

However, little is known about LR in teleosts at present. Owing to the poor conservation of the leptin amino acid sequences between mammals and teleosts, the first teleostean leptin was cloned more than a decade after it was first cloned in mammals (11). Nonetheless, strong conservation of the tertiary structure throughout the vertebrates and the same anorexigenic effect upon leptin administration suggested the conservation of leptin action (12). Unlike in mammals, in which adipose tissue is the primary site for leptin synthesis, in fish leptin was reported to be mainly expressed in the liver (8). Furthermore, owing to the less pronounced lipolytic action of teleostean leptin coupled with its clearer glycometabolic action, some researchers demonstrated that teleostean leptin may play a greater role as a glucoregulatory hormone than an adipostatic factor (10, 13). However, the roles of leptin in fish which under over-nutrition statuses are still unclear.

In this study, we fed Nile tilapia (*Oreochromis niloticus*), which is an ideal fish model for nutritional and metabolic studies (14), a high-carbohydrate diet (HCD) or a high-fat diet (HFD) and subjected it to Nile tilapia recombinant leptin A (NtrelepA) treatment. The aims were (i) to determine the function of leptin in Nile tilapia, (ii) to verify the existence of LR in Nile tilapia, and (iii) to assess the action of leptin in DIO Nile tilapia. For the first time, our study confirms the existence of LR in DIO Nile tilapia and demonstrates a selective leptin action in nutrients metabolism in HCD-DIO Nile tilapia and HFD-DIO Nile tilapia which could be an adaptive strategy for surplus calorie storage.

## Materials and methods

### Experimental animals

All-male Nile tilapia were purchased from Bairong Fish Farm in Qingyuan (Guangdong, China) and were maintained at 28 ± 1°C in the circulating water system with a 12D:12L photoperiod. The fish were acclimated for 1 week before the study. All experiments were conducted under the Guidance of the Care and Use of Laboratory Animals in China. This research was approved by the Committee on the Ethics of Animal Experiments of East China Normal University.

### Preparation of NtrelepA

Nile tilapia has two isoforms of leptins namely; leptin A and leptin B. Leptin A (LepA), which is the dominant leptin ortholog (15) was used in this study. NtrelepA was expressed in the *Trans*B (DE3) chemically competent cell (TransGen, China). A pair of primers (Table S1) was used to obtain the fragment encoding the mature protein of Nile tilapia Leptin A (LepA) (Ala21-Cys161) with 6 tandem histidine at the N-terminal. The fragment was digested by using NdeI and XhoI (TaKaRa, Japan) before it was cloned into the same restriction-enzyme sites in pET-32a (+) (Novagen, Germany). Heat shock method was used to transform the recombinant plasmid into *Trans*B (DE3) chemically competent cell for protein expression. The Ni-NTA column (Yelibio, China) was used to purify the fusion protein and the purified buffer was replaced with saline buffer by dialysis membranes (Yuanyebio, China). The purified protein was detected by sodium dodecyl sulfate-polyacrylamide gel electrophoresis (SDS-PAGE) and was identified by matrix-assisted laser desorption/ionization-time of flight mass spectrometry (MALDI-TOF-MS) (Figures S1A,B). The concentration of NtrelepA was determined by the bicinchoninic acid (BCA) kit (KWBIO, China). The activity of NtrelepA was determined by intraperitoneal injection (i.p.) of NtrelepA in Nile tilapia. The blood glucose level increased gradually with an increase of NtrelepA concentration, suggesting the normal activity of NtrelepA (Figure S1C).

### Fish feeding and intraperitoneal (i.p.) injections

The fish used for LepA expression profile and NtrelepA i.p. injection were fed twice daily to visual satiation by using a commercial diet (Tongwei, China) for 4 weeks (final weight 13 ± 2 g). After fasting the fish for overnight, foregut, spleen, heart, kidney, visceral adipose tissue (VAT), red muscle, white muscle, brain, gill, skin and liver were collected for LepA expression profile. To assess the *in vivo* regulation of NtrelepA in appetite and metabolism, Nile tilapia were divided into two groups and each group received a single i.p. injection of NtrelepA (400 ng/g BW) or saline (30 fish each group). The fish were given enough feeds and allowed to consume the feeds for 15 min at 1, 2, 4, 7, 11 h after administration. During this period, the amount of food intake at each time point was recorded and the cumulative food intake during the 11 h in both groups were calculated and compared. Two hours after administration of NtrelepA or saline, six unfed fish in each group were removed to collect blood, liver, hypothalamus and muscle for biochemical or molecular assays.

In another experiment, some Nile tilapia were used to establish DIO models. Fish were fed a normal diet (ND; 4% fat and 32.9% corn starch), a high-carbohydrate diet (HCD; 4% fat and 44.9% corn starch), or a high-fat diet (HFD; 16% fat and 32.9% corn starch) (Table S2). Body weights of the fish in each treatment were measured weekly. The fish were fed on the respective diets daily at a feeding rate of 5% body weight for 8 weeks. To investigate the physiological changes of the DIO models, six fish from each diet were fasted for overnight and sacrificed by anesthetizing them with MS-222 (0.1 g/L). The liver and VAT were collected and weighed, and the muscle, hypothalamus, subcutaneous adipose tissue (SCAT) and plasma were collected from each sacrificed fish. To determine the actions of leptin in food intake regulation under DIO states, 12 fish were sampled from each dietary treatment and divided into two groups. One group received an i.p. injection of NtrelepA (400 ng/g BW) while the other group was i.p. injected with saline. Two hours after injection, the fish were fed a single meal with enough feeds and the food intake was counted after 15 min.

To monitor the weight of nine fish from each dietary treatment received an i.p. administration of NtrelepA (400 ng/g BW) or saline. Two hours after injection, fish were given a single meal with enough feed and the weight gain was calculated after 24 h of injection. For metabolic assays, 12 fish from each dietary treatment were divided into two groups. Each group received an i.p. injection of NtrelepA (400 ng/g BW) or saline. Two hours after treatment, plasma, liver, hypothalamus and muscles were collected. To study the action of leptin restoration, 12 fish from each dietary group were deprived of feeds for 1 week followed by feeding for 8 weeks. The fish were then divided into two groups and were i.p. injected with NtrelepA (400 ng/g BW) or saline. Two hours after treatment, plasma, liver and muscles were collected. All fish were anesthetized with MS-222 (0.1 g/L) before sampling and the samples were immediately frozen in liquid nitrogen, followed by storage at-80°C until used.

### Quantitative real-time PCR

Total RNA was isolated using TransZol^TM^ (TransGen, China) and reverse-transcribed using the PrimeScript^TM^ RT Master Mix Kit (Takara, Japan). qPCR was performed in a CFX96 real-time PCR system (Bio-Rad, USA) using SYBR Mix (KWBIO, China). Pre-validated primers spanning exon-exon boundaries were used for amplifications and each PCR run was performed in duplicate. Elongation factor 1 alpha (*EF1a*) was used as reference gene. All primer sequences are listed in Table S1.

### Fatty acid β-oxidation

The fatty acid (FA) β-oxidation assay was performed as previously reported in our previous study (16). The Nile tilapia liver tissues treated with NtrelepA or saline were weighed and homogenized (1:40, w/v) in ice-cold 0.25 M-sucrose medium containing 2 mM-EGTA and 10 mM-Tris-HCl, pH 7.4. The homogenates were used for immediate measurements of [1-^14^C] palmitic acid (PerkinElmer) β-oxidation. Tri-Carb 4910TR Liquid Scintillation Analyzer (PerkinElmer) was used for radioactivity measurements.

### [1-^14^C] D-glucose tracer assay

A sample of six fish (about 13 ± 2 g) from each treatment group were starved for overnight and first received i.p. injection of NtrelepA or saline, followed by i.p. injection of [1-^14^C] D-glucose (50 μCi/kg BW, PerkinElmer) 1 hour after the first injection. One hour after the second injection, the whole fish was digested by using a digestion solvent (70% HClO_4_/30% H_2_O_2_, v/v) (1:5, w/v) at 6°C in a water bath for 12 h. The radioactivity of whole fish was measured in Tri-Carb 4910TR Liquid Scintillation Analyzer (PerkinElmer).

### Glucose/insulin tolerance tests

For intraperitoneal glucose tolerance test (GTT), fish were fasted for overnight, followed by an i.p. injection of D-glucose (500 mg/kg BW, 20% in 0.85% NaCl) (Sigma, Germany). Plasma glucose levels were measured using a glucose assay kit (Jiancheng, China) at 0, 30, 90, and 180 min after injection. For one time point GTT with NtrelepA administration, fish received NtrelepA administration first, then i.p. injection of D-glucose 1 h after NtrelepA injection. Plasma glucose levels were determined 1 h after D-glucose injection. For intraperitoneal insulin tolerance test (ITT), fish were fasted for 4 h and were i.p. injected with bovine insulin (0.75 U/kg BW) (Sigma, Germany). Glucose levels were determined 90 min after injection as described above.

### Histological and cytological analyses

Liver, SCAT (including muscle and skin), and VAT (at least four fish from each group) were fixed in 4% paraformaldehyde (Servicebio, China) and embedded in paraffin as described previously (17). Paraffin blocks were sectioned at 6 μm thickness using a microtome, followed by hematoxylin and eosin (H & E) (Sigma). The H & E staining was carried out following a standard procedure Reference(s). For immunohistochemistry (IHC) staining of liver and SCAT, paraffin-sectioned samples were pretreated in citrate buffer and digested by using microwave oven for antigen repair in 3% H_2_O_2_ for 25 min and blocked in 3% BSA for 30 min. LepA-specific rabbit polyclonal antibody (HuaAn, China) was added in the blocking solution (1:200) and incubated at 4°C for overnight. Sections were washed with phosphate buffered saline (PBS) and incubated with goat anti-rabbit IgG-HRP (HuaAn, China) secondary antibody for 1 h at room temperature. The signal was detected by 3,3′-diaminobenzidine (DAB) kit (Servicebio, China).

### Measurements of hormones and metabolites

Enzyme-linked immunosorbent assay (ELISA) kits were used to measure plasma insulin, glucagon and leptin levels (mlbio, China). The plasma glucose, triglyceride (TG), non-esterified fatty acid (NEFA), hepatic TG and glycogen and muscle glycogen were determined by colorimetric enzymatic assays (Jiancheng, China), according to the manufacturer's protocols. Total lipid contents (percent dry weight) in whole Nile tilapia were measured using the Folch procedure as previously described (18).

### Western blot analysis and antibodies

Protein extraction and SDS-PAGE were performed as described previously (14). Rabbit polyclonal antibody, β-actin and GAPDH were purchased from SunGene Biotech (SunGene, China). LepA-specific rabbit polyclonal antibody was obtained from HuaAn Biotech (HuaAn, China). Signal transducers and activators of transcription 3 (STAT3) and phosphorylation of STAT3 (p-STAT3) rabbit polyclonal antibodies were purchased from Elabscience Biotech (Elabscience, China).

### Transcriptomic assay

Nine hepatic RNA samples (three from each dietary treatment group) were prepared after feeding trial. The libraries were deep sequenced with the Illumina Hiseq 2500 platform. The raw reads were filtered for quality control as described previously (19). The whole set of original sequences are available in the NCBI Short Read Archive under accession number SRP120142.

### Differentially expressed genes (DEGs) and function enrichment analyses

Clean RNA-seq reads were mapped to the Nile tilapia reference genome. The expression level of transcripts was normalized using fragments per kilobase of transcript per million mapped (FPKM) reads method. DEGs analysis across groups was determined using edgeR package (http://www.rproject.org/). In the present study, the genes with fold change ≥2 and FDR ≤ 0.05 were deemed to be the significant DEGs. Gene Ontology (GO) and Kyoto Encyclopedia of Genes and Genomes (KEGG) enrichment analysis were conducted basing on the DEGs.

### Statistical analysis

Results are presented as mean ± SEM. Data were tested for normality using Shapiro–Wilk test and homogeneity of variance using Levene's test. Comparisons between two groups were performed by using two-tailed independent *t*–test. Results with *P* < 0.05 were considered to be significant. Analyses were performed using GraphPad Prism (GraphPad, San Diego, California, USA).

## Results

### LepA expression profile in Nile tilapia

**Figure 1:**
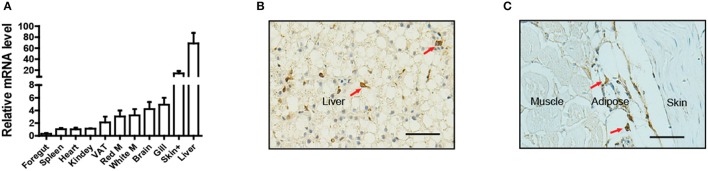
Expression profile of LepA in Nile tilapia. **(A)** *LepA* mRNA levels in 11 tissues. White M (white muscle), Red M (red muscle), VAT (visceral adipose tissue), Skin+ (skin and subcutaneous adipose tissue), (*n* = 3). **(B)** IHC for LepA (brown, indicated by red arrows) in liver. Scale bars, 50 μm. **(C)** IHC for LepA (brown, indicated by red arrows) in SCAT. Scale bars, 50 μm.

To determine the tissue distribution of *LepA* in Nile tilapia, a total of 11 tissues were sampled for qPCR analysis. Consistent with the findings of many studies in fish, the highest *LepA* mRNA expression levels were obtained in the liver (Figure 1A). Surprisingly, the organ with the second highest expression was the skin. Further analysis by using IHC with a LepA-specific antibody indicated that LepA is expressed in liver and SCAT (Figures 1B,C). These results confirm that SCAT is another site which has high expression of LepA.

### LepA regulates food intake and activates catabolism in Nile tilapia

**Figure 2:**
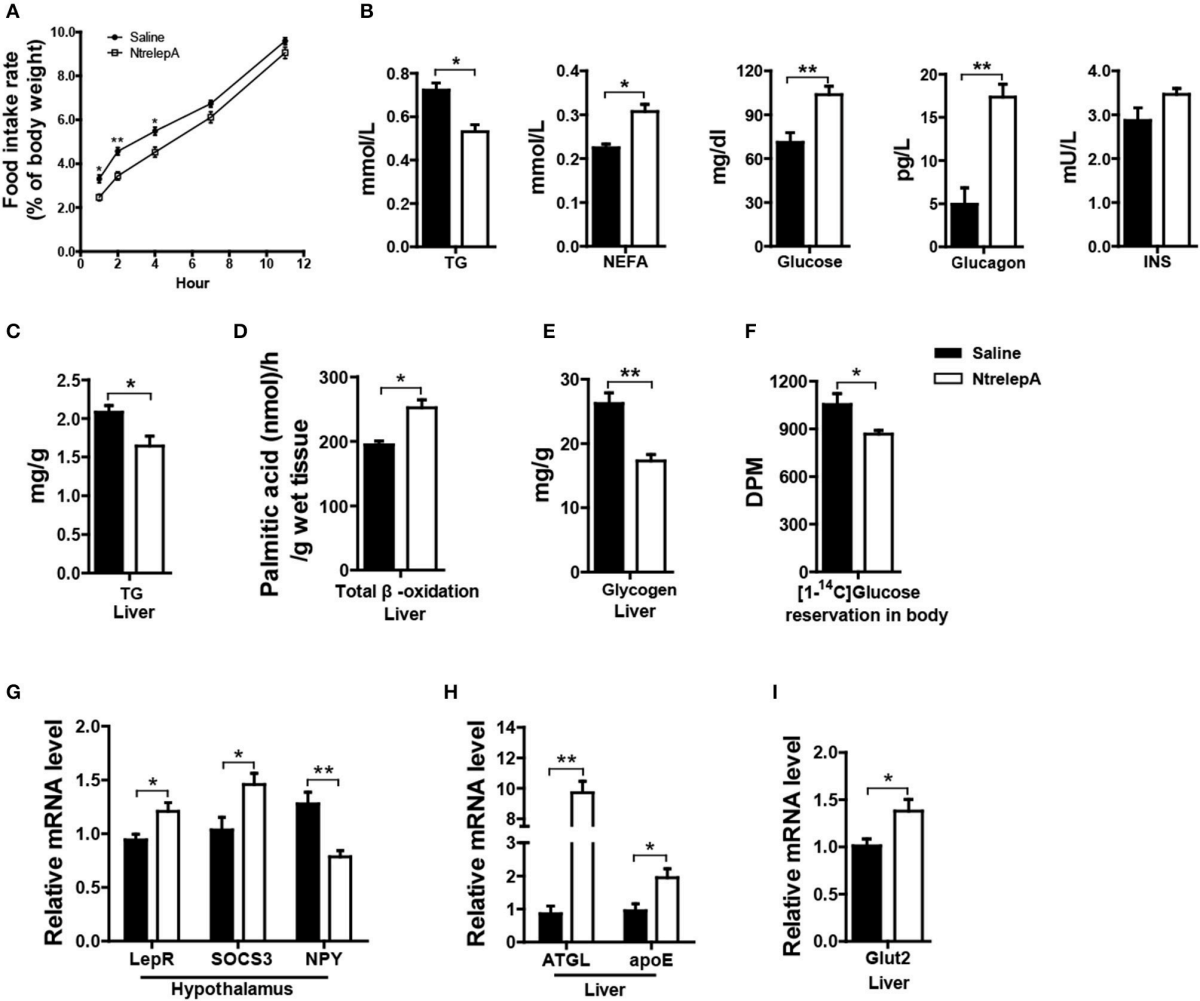
Physiological functions of LepA in Nile tilapia. **(A)** Accumulated food intake rate, (*n* = 3, 5 fish each parallel). **(B)** Plasma TG (*n* = 6), NEFA (*n* = 6), glucose (*n* = 7), glucagon and insulin (INS) levels (*n* = 5). **(C)** Liver TG content (*n* = 6). **(D)** Total β-oxidation of [1-^14^C] palmitate in the liver homogenates (*n* = 6). **(E)** Liver glycogen content (*n* = 6). **(F)** Reservation of [1-^14^C] D-glucose in body (*n* = 6). **(G)** *LepR, SOCS3* and *NPY* mRNA in hypothalamus (*n* = 6). **(H)** *ATGL* and *apoE* mRNA in liver (*n* = 6). **(I)** *Glut2* mRNA in liver (*n* = 6). *p < 0.05 and ^**^p < 0.01. Data shown as means ± SEM.

To investigate whether LepA has an anorexic effect and activates catabolism, we examined the physiological responses to acute leptin treatment. NtrelepA was obtained by an *E. coli*-derived method. We conducted intraperitoneal (i.p.) injection of NtrelepA (400 ng/g BW) (20) and the food intake at 1, 2, 4, 6, 7, 11 h after NtrelepA treatment were measured. The final cumulative food intake was reduced significantly after 1–7 h after NtrelepA treatment (Figure 2A). This confirms that LepA has an acute anorexic effect in Nile tilapia.

Furthermore, 2 h after NtrelepA treatment, the plasma TG levels decreased, and the NEFA, glucose, and glucagon levels increased, while there was no change in insulin levels (Figure 2B). As a response to these changes, liver TG content was decreased and FA β-oxidation in liver was enhanced (Figures 2C,D). In addition, liver glycogen content decreased and the reservation of injected [^14^C] glucose in the body also decreased (Figures 2E,F).

To better understand the molecular basis of these effects, we analyzed the expression of genes involved in appetite and metabolism under NtrelepA treatment. Consistent with previous report (21), the hypothalamic leptin signaling pathway was activated by inducing leptin receptor (*LepR*) mRNA expression, decreasing *NPY* mRNA expression, and also promoting mRNA expression of the negative feedback regulator, *SOCS3* (Figure 2G). The mRNA levels of genes involved in lipolysis (*ATGL*) and lipid transport (*apoE*), were significantly increased in the liver after NtrelepA treatment (Figure 2H). NtrelepA also increased the hepatic expression of *Glut2*, the key glucose transporters (Gluts) in whole-body glucose utilization (22) (Figure 2I). These findings suggest that LepA activates the catabolism of lipid and glucose.

### Production of DIO Nile tilapia model

**Figure 3:**
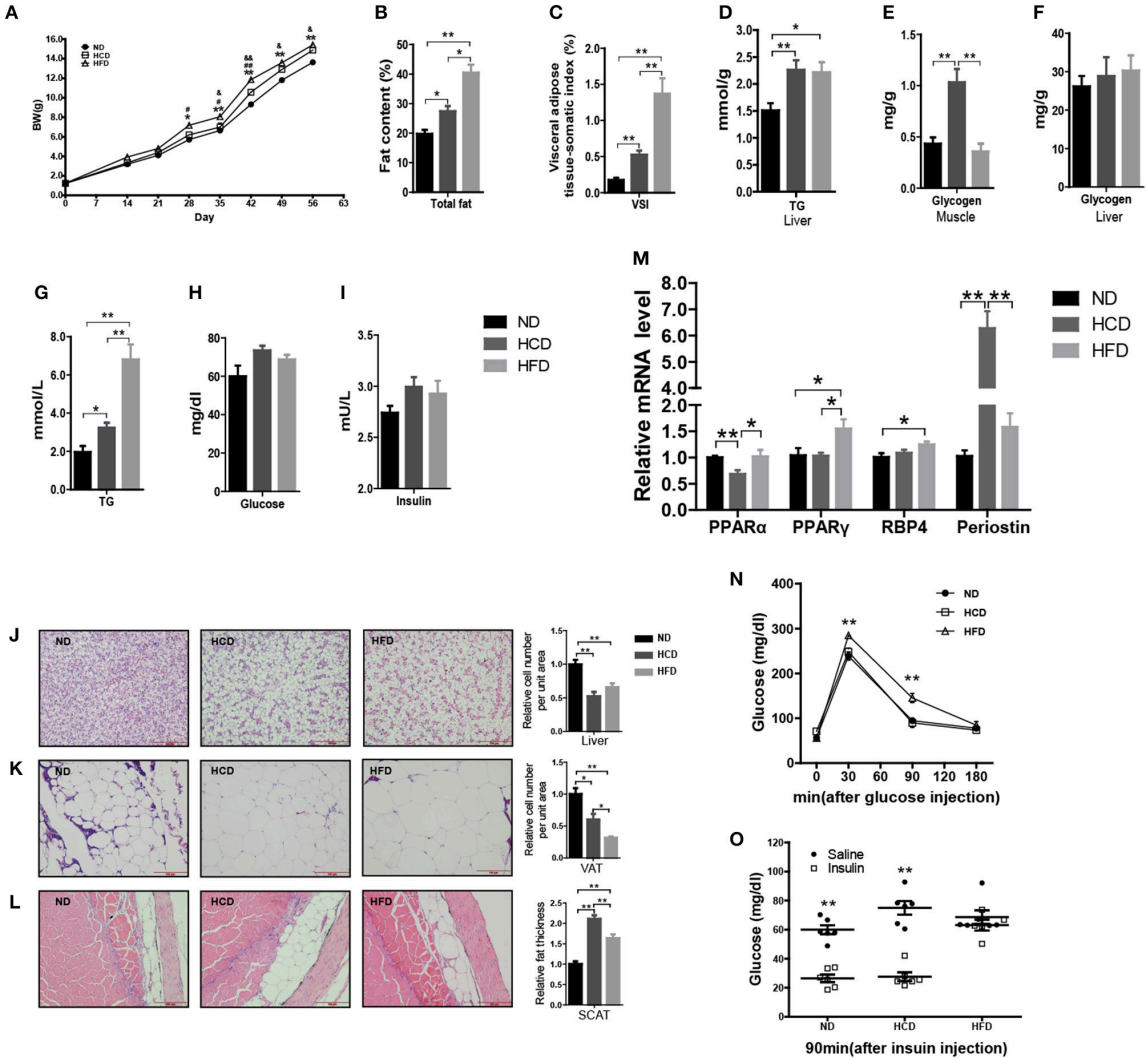
Body composition and metabolic characteristics of DIO Nile tilapia induced by HCD or HFD feeding. **(A)** Body weight changes. ^#^HFD vs. HCD, ^&^HCD vs. ND, ^#^*P* < 0.05, ^&^*P* < 0.05, ^*##*^*P* < 0.01 and ^&&^*P* < 0.01 (*n* = 3, 120 fish per group). **(B)** Whole fish fat content (100% × fat weight/body weight, dry weight ratio) (*n* = 6). **(C)** Visceral adipose tissue-somatic index (VSI) (100 × visceral adipose tissue weight / body weight, wet weight ratio) (*n* = 6). **(D)** Liver TG content (*n* = 6). **(E)** Muscle glycogen content (*n* = 6). **(F)** Liver glycogen content (*n* = 6). **(G–I)** Plasma TG, glucose and insulin levels (*n* = 6). **(J–L)** H&E staining of liver, VAT and SCAT. Three column graphs in the right of pictures from top to bottom represent hepatocyte size, adipocyte size and thickness of SCAT, respectively. Scale bars, 100 μm. **(M)** *PPAR*α and *PPAR*γ mRNA levels in liver (*n* = 6). **(N,O)** GTT (*n* = 5) and ITT (*n* = 6). ^*^p < 0.05 and ^**^p < 0.01. Data shown as means ± SEM.

In mammals, LR is tightly correlated with obesity. To produce the DIO Nile tilapia model and also evaluate the roles of hyperleptinemia in different nutritional statuses, we fed the fish a diet containing either 44.9% corn starch (HCD) or 16% fat (HFD), and 4% fat with 32.9% corn starch as a normal diet (ND). After 8 weeks of feeding, the mean body weights of the fish in the HCD and HFD groups were 9 and 13% higher than that of the ND-fed fish, respectively (Figure 3A). Correspondingly, significantly higher fat deposition in whole body, liver, VAT, and SCAT was obtained in the HCD- and HFD-fed fish than those in the control (Figures 3B,C,J–L). Compared to HCD, HFD-fed fish had higher fat deposition (Figures 3B,C). Notably, HCD induced a larger mass of SCAT than HFD (Figure 3L). Both HCD and HFD increased significantly hepatic TG content and plasma TG levels (Figures 3D,G). More muscle glycogen was accumulated in HCD-fed fish, however no significant differences were observed among three groups in plasma glucose, insulin and hepatic glycogen (Figures 3E,F,H,I). Moreover, HCD decreased *PPAR*α mRNA levels and increased expression of *periostin*, while HFD increased *PPAR*γ and *RBP4* mRNA levels in liver (Figure 3M). Additionally, GTT and ITT showed that HFD induced insulin resistance while HCD-fed fish had normal insulin sensitivity (Figures 3N,O).

**Figure 4:**
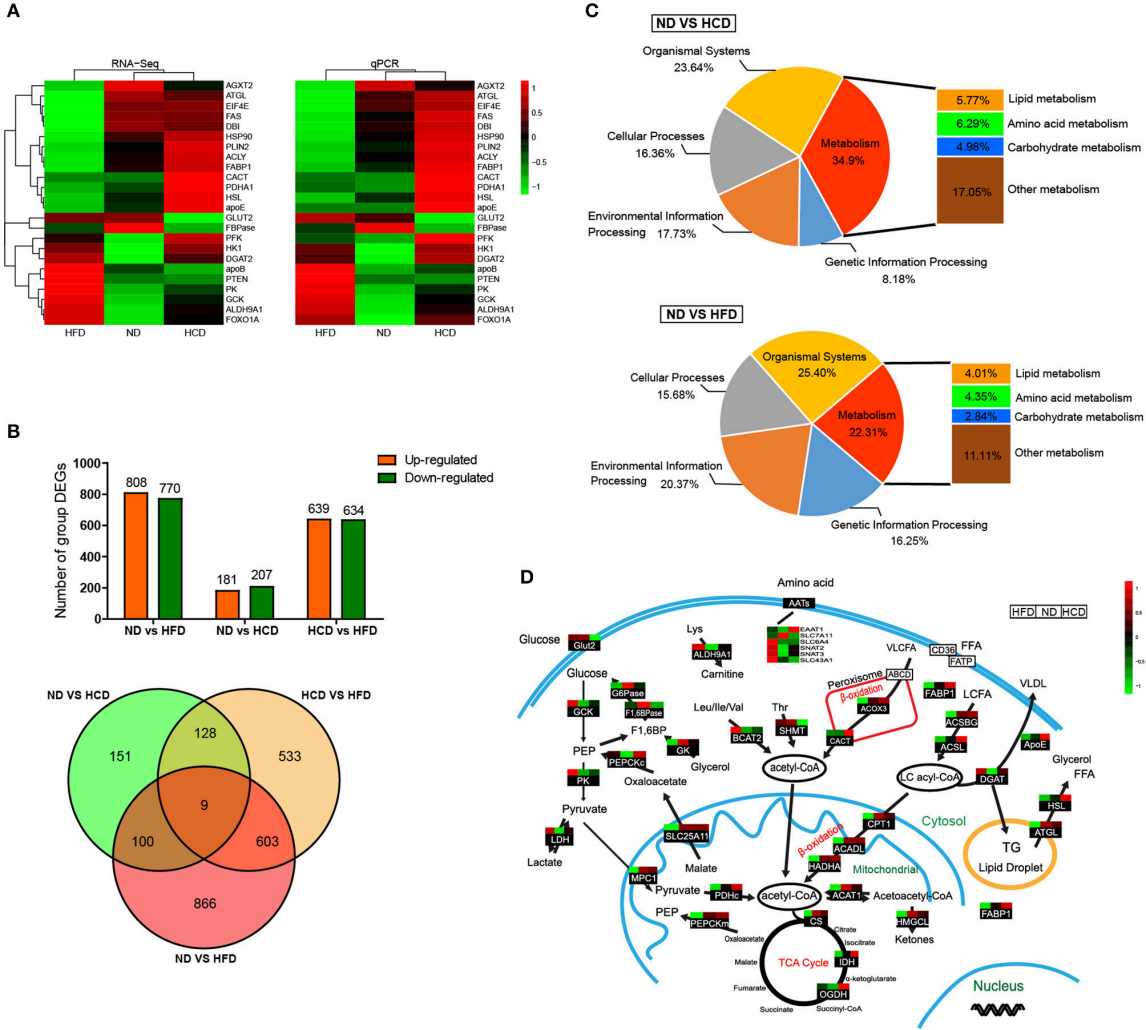
Comparative transcriptomic analysis of HCD or HFD-induced obese Nile tilapia. **(A)** Validation of RNA-Seq data by qRT-PCR. The left profile shows mRNA expression patterns of 24 representative genes detected by RNA-Seq, and the right one is the corresponding verification results by qRT-PCR. *EF1*α was used as an internal control. **(B)** The number of DEGs among all samples. **(C)** Distribution by categories of DEGs when compared HCD and HFD treatment to control. FDR ≤ 0.05 and |log_2_ (fold change)| ≥1 were set as the thresholds to identify DEGs. **(D)** DEGs involved in metabolic pathways of DIO Nile tilapia. Relative expression levels were normalized based on the Z-score and are shown on a color gradient from low (green) to high (red). GCK, glucokinase; PK, pyruvate kinase; LDH, lactate dehydrogenase; G6Pase, glucose 6-phosphatase; F1,6BPase, fructose 1,6-bisphosphatase; PEPCKc, phosphoenolpyruvate carboxykinase cytosol isozyme; GK, glycerol kinase; ALDH9A1, aldehyde dehydrogenase 9 family, member A1; BCAT2, branched-chain-amino-acid aminotransferase; SHMT, serine hydroxymethyltransferase; EAAT1, excitatory amino acid transporter 1; SLC7A11, solute carrier family 7 member 11; SLC6A4, solute carrier family 6 member 4; SNAT2, sodium-coupled neutral amino acid transporter 2; SNAT3, sodium-coupled neutral amino acid transporter 3; SLC43A1, solute carrier family 43 member 1; ACOX3, acyl-coenzyme A oxidase 3; CACT, carnitine-acylcarnitine translocase; FABP1, fatty acid-binding protein 1; ACSBG, acyl-CoA synthetase, bubblegum Family; ACSL, long chain fatty acid-CoA ligase; ApoE, apolipoprotein E; DGAT, diglyceride acyltransferase; HSL, hormone-sensitive lipase; ATGL, adipose triglyceride lipase; MPC1, mitochondrial pyruvate carrier 1; SLC25A11, solute carrier family 25 member 11; PDHc, pyruvate dehydrogenase complex; PEPCKm, phosphoenolpyruvate carboxykinase mitochondrial isozyme; CPT1, carnitine palmitoyl transterase 1; ACADL, acyl-CoA dehydrogenase long chain; HADHA, hydroxyacyl-Coenzyme A dehydrogenase; ACAT1, acetyl-CoA acetyltransferase 1; HMGCL, hydroxymethylglutaryl-CoA lyase; CS, citrate synthase; IDH, isocitrate dehydrogenase; OGDH, ketoglutarate dehydrogenase.

To systemically elucidate the metabolic characteristics of DIO fish, we carried out global transcriptome analysis. The well-matched RNA-Seq and qPCR analyses of the expression of 24 genes indicated the reliability of the RNA-Seq data (Figure 4A). There were 1,578 different expression genes (DEGs) in HFD, but only 388 DEGs in HCD, when compared to ND. Moreover, there were only nine common DEGs in the three pairwise groups (Figure 4B). Classification analysis showed that the pathway that was predominantly affected by the HCD was “metabolism” while that by the HFD was “organismal systems” (Figure 4C). We also created a schematic illustration of the DEGs involved in the major metabolic pathways. Interestingly, HCD-fed fish showed more activity in lipid catabolism, while HFD-fed fish were more active in glucose catabolism (Figure 4D). Taken together, these findings indicate that both HCD and HFD induced DIO Nile tilapia, however, the two DIO models had distinct metabolic characteristics.

### Leptin resistance occurred in DIO Nile tilapia

**Figure 5:**
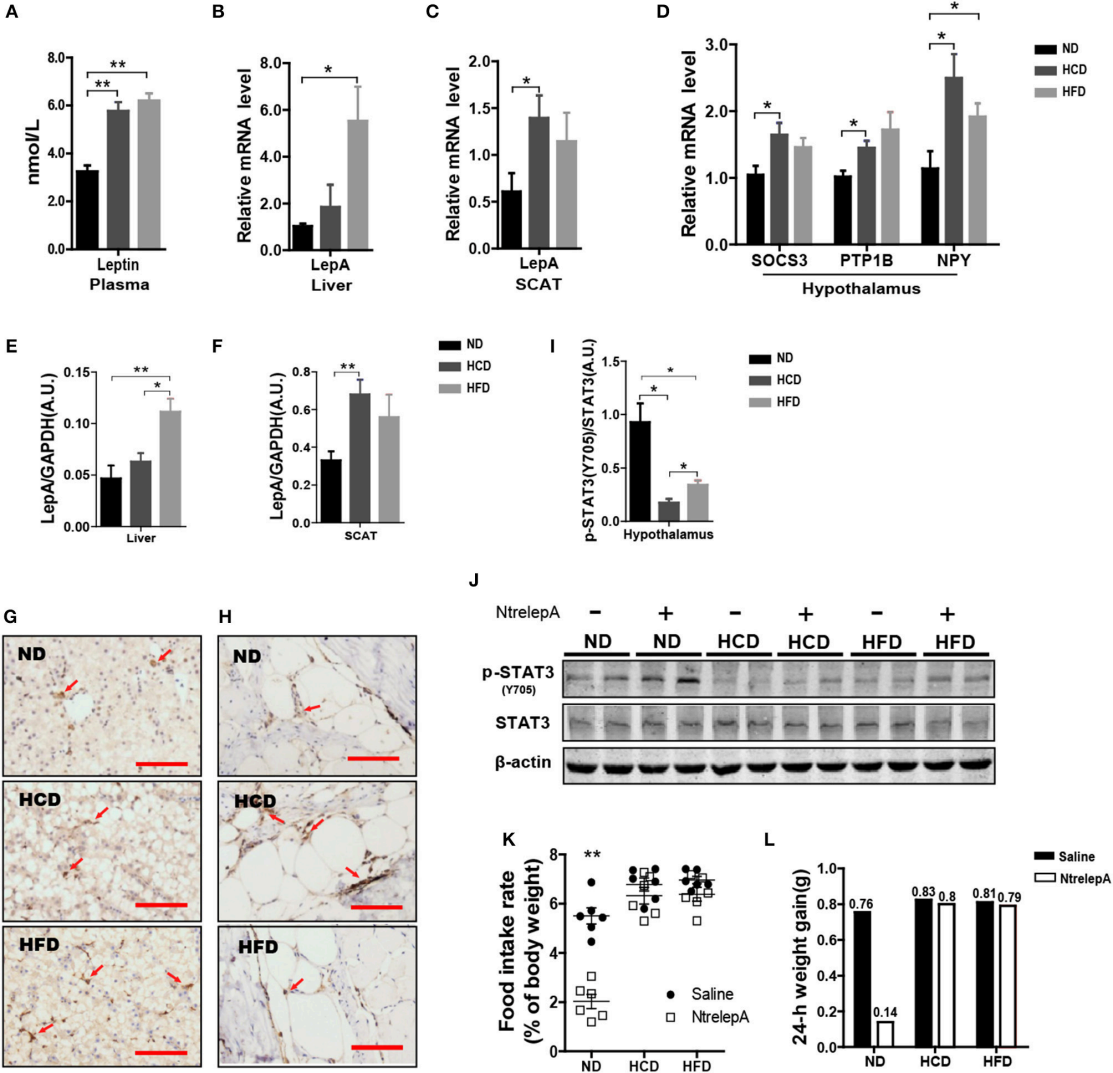
DIO Nile tilapia presented hyperleptinemia and LR. **(A)** Plasma Leptin levels (*n* = 5). **(B,C)** *LepA* mRNA levels in liver and SCAT (*n* = 6). **(D)** *SOCS3, PTP1B* and *NPY* mRNA levels in hypothalamus (*n* = 6). **(E,F)** Western blot analysis of LepA in liver and SCAT, and column diagram shows as LepA relative to GAPDH (*n* = 4). **(G,H)** IHC for LepA (brown, indicated by red arrows) in liver and SCAT. Scale bars, 50 μm. **(I)** Western blot analysis of p-STAT3 in hypothalamus, and column diagram is shown as p-STAT3 relative to STAT3 (*n* = 4). **(J)** Hypothalamic p-STAT3 with NtrelepA administration. **(K)** Food intake rate at 2 h after NtrelepA or saline treatment (*n* = 6). **(L)** 24-h weight gain after NtrelepA or saline treatment. NtrelepA group (9 ND fish, 8 HCD fish, and 9 HFD fish) and saline group (10 ND fish, 8 HCD fish, and 10 HFD fish). ^*^p < 0.05 and ^**^p < 0.01. Data shown as means ± SEM.

Hyperleptinemia is an important characteristic of DIO mice (23). However, little is known about the expression of leptin in DIO teleosts. Consistent with the findings in DIO mice, Nile tilapia fed HCD or HFD showed elevated plasma leptin (Figure 5A). To find out the source of the plasma leptin, we determined the mRNA expression of *LepA* in liver and SCAT, and found that higher *LepA* mRNA expression in HFD- and HCD-fed fish occurred in liver and SCAT, respectively (Figures 5B,C). This was confirmed by western blotting and IHC (Figures 5E–H). Thus, DIO Nile tilapia showed hyperleptinemia, but the main sites of LepA synthesis differed between HCD- and HFD-DIO fish.

In mammals, hyperleptinemia involves enhancement of the leptin signaling pathway, but this is weakened in DIO mice (24). Accordingly, we examined the genes at downstream of leptin signal transduction and p-STAT3 in the hypothalamus. Feeding fish with HCD or HFD, and especially the former, induced both *SOCS3* and *PTP1B* mRNA expression in the hypothalamus (Figure 5D). Accordingly, the expression of *NPY* mRNA was increased in HCD- and HFD-fed fish (Figure 5D). Similarly, levels of p-STAT3 decreased significantly in HCD- and HFD-fed fish, but this was more pronounced in HCD-fed fish (Figure 5I). Moreover, NtrelepA administration increased p-STAT3 in ND-fed fish, but this effect was attenuated in HCD- and HFD-fed fish (Figure 5J). Thus, DIO Nile tilapia showed a hyperleptinemic state with impaired leptin signaling.

Based on this, we examined the *in vivo* effects of peripheral leptin on food intake and 24 h-weight gain after NtrelepA injection. NtrelepA treatment clearly decreased food intake and weight gain in ND fish, but these effects were eliminated in both HCD and HFD groups (Figures 5K,L). Hyperleptinemic DIO mice with blunted anorectic responsiveness to exogenous leptin administration are commonly referred to as exhibiting LR (3). Our results indicate for the first time that LR occurs in obese Nile tilapia upon feeding on an HCD or HFD for 8 weeks.

### DIO Nile tilapia selectively retain leptin action in metabolic regulation

**Figure 6:**
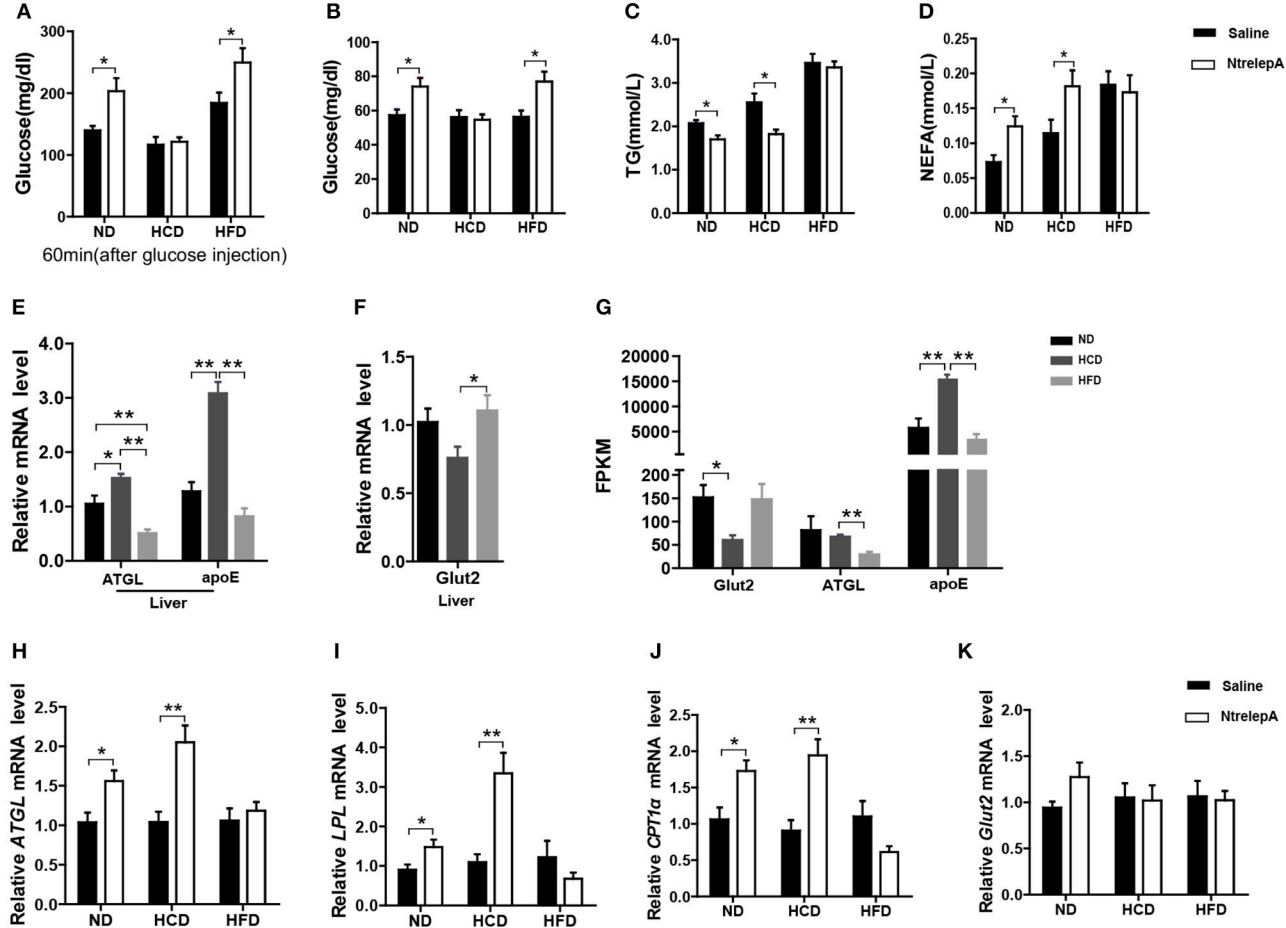
DIO Nile tilapia with LR presented selective regulation of leptin in lipid or glucose metabolism**. (A)** NtrelepA administration effects on glucose tolerance test (*n* = 5). **(B–D)** NtrelepA administration effects on plasma glucose, TG and NEFA levels (*n* = 6). **(E)** *ATGL* and *apoE* mRNA in liver (*n* = 6). **(F)** *Glut2* mRNA in liver (*n* = 6). **(G)** FPKM of *Glut2, ATGL*, and *apoE* in liver. **(H–K)** Gene expression analysis 2 h after NtrelepA administration (*n* = 6). *ATGL* mRNA levels in liver **(H)**. *LPL* and *CPT1a* mRNA levels in liver **(I,J)**. *Glut2* mRNA levels in liver **(K)**. ^*^p < 0.05 and ^**^p < 0.01. Data shown as means ± SEM.

To test whether leptin retains its actions in metabolic regulation in DIO fish, NtrelepA injection was conducted in fish after 8 weeks of experimental feeding. The results showed that NtrelepA treatment increased the plasma glucose level in ND- and HFD-fed fish, but it remained unchanged in HCD-fed fish, in either a GTT or a normal state (Figures 6A,B). In contrast, NtrelepA treatment decreased plasma TG and increased plasma NEFA in ND- and HCD-fed fish, but did not change these parameters in HFD-fed fish (Figures 6C,D). The results demonstrate that both HCD and HFD induced LR, but the regulatory action of leptin was selectively retained in lipid metabolism in HCD-DIO fish and in glucose metabolism in HFD-DIO fish.

### Selectively-retained leptin action is beneficial for surplus calories storage

**Figure 7:**
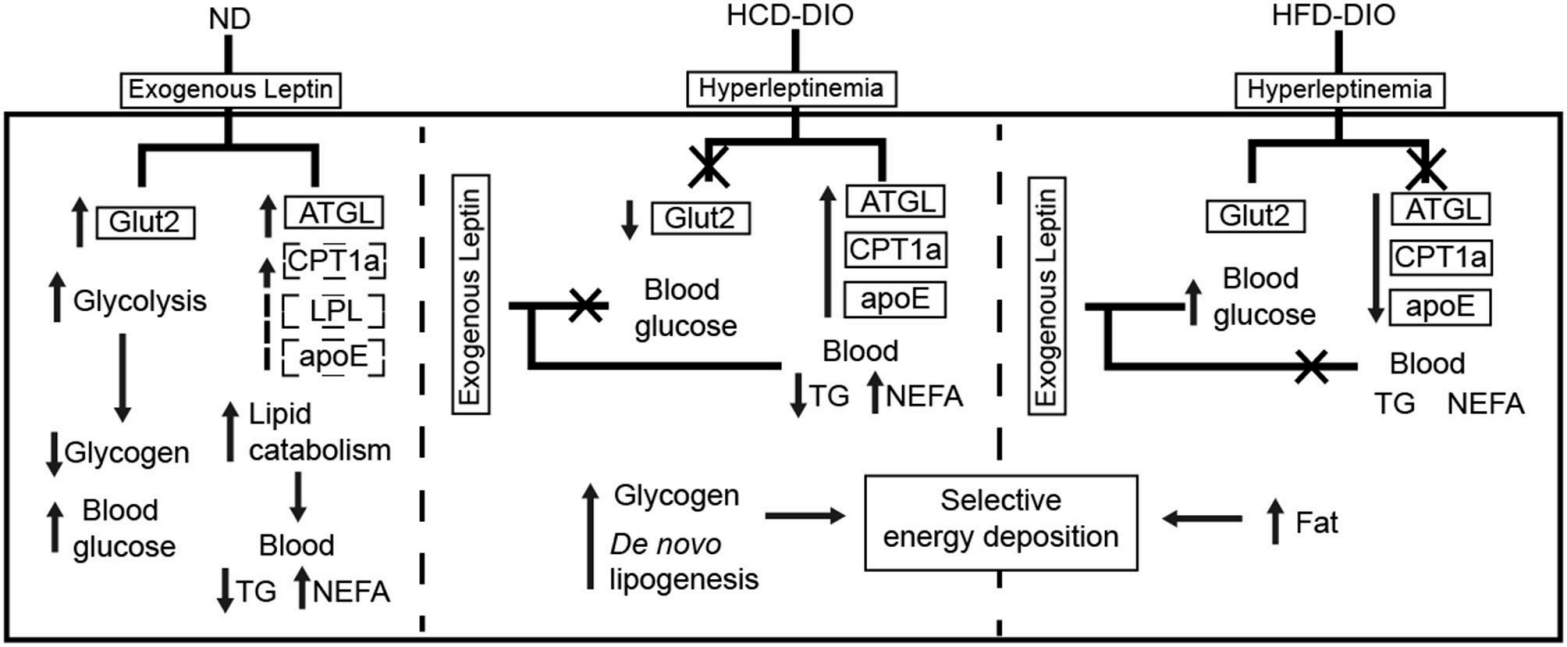
Diagram of selective metabolic regulation of leptin in DIO Nile tilapia induced by HCD or HFD feeding.

To clarify the selective regulatory actions of leptin in metabolism in DIO fish, we determined the mRNA expression of leptin-regulated genes under hyperleptinemic states by qPCR. Interestingly, HCD-fed fish exhibited increased *ATGL* and *apoE* expression in liver, but had a decreased *Glut2* expression in the same tissue. In contrast, HFD-fed fish exhibited decreased *ATGL* and *apoE* expression, but maintained the expression of *Glut2* in the liver (Figures 6E,F). These findings were confirmed by transcriptomic analysis (Figure 6G). Next, we determined the expression of these genes upon NtrelepA administration. The mRNA levels of lipid metabolism-related genes, such as *ATGL, LPL*, and *CPT1a*, were increased by NtrelepA in ND- and HCD-fed fish, but remained unchanged in HFD-fed fish (Figures 6H–J), suggesting LepA abated the ability to regulate lipid metabolism. Fish fed with ND and those treated with NtrelepA had an increased expression of *Glut2* mRNA in the liver, but no change was obtained in HCD-fed fish (Figure 6K), suggesting that LepA weakened the regulation of glucose metabolism in HCD-fed fish. These findings provide biochemical and molecular evidences that HCD-DIO fish retain the action of leptin in lipid metabolism, while HFD-DIO fish retain its action in glucose metabolism. This selective LR could be correlated with a previous theory that HFD-induced obesity required the blockade of leptin action for surplus calorie storage (25). Accordingly, we generated a diagram to reveal the selective blockade of leptin action in metabolic regulation when obesity was induced in fish by feeding on an HCD or HFD (Figure 7).

### Fasting for 1 week restores leptin regulation in DIO Nile tilapia

**Figure 8:**
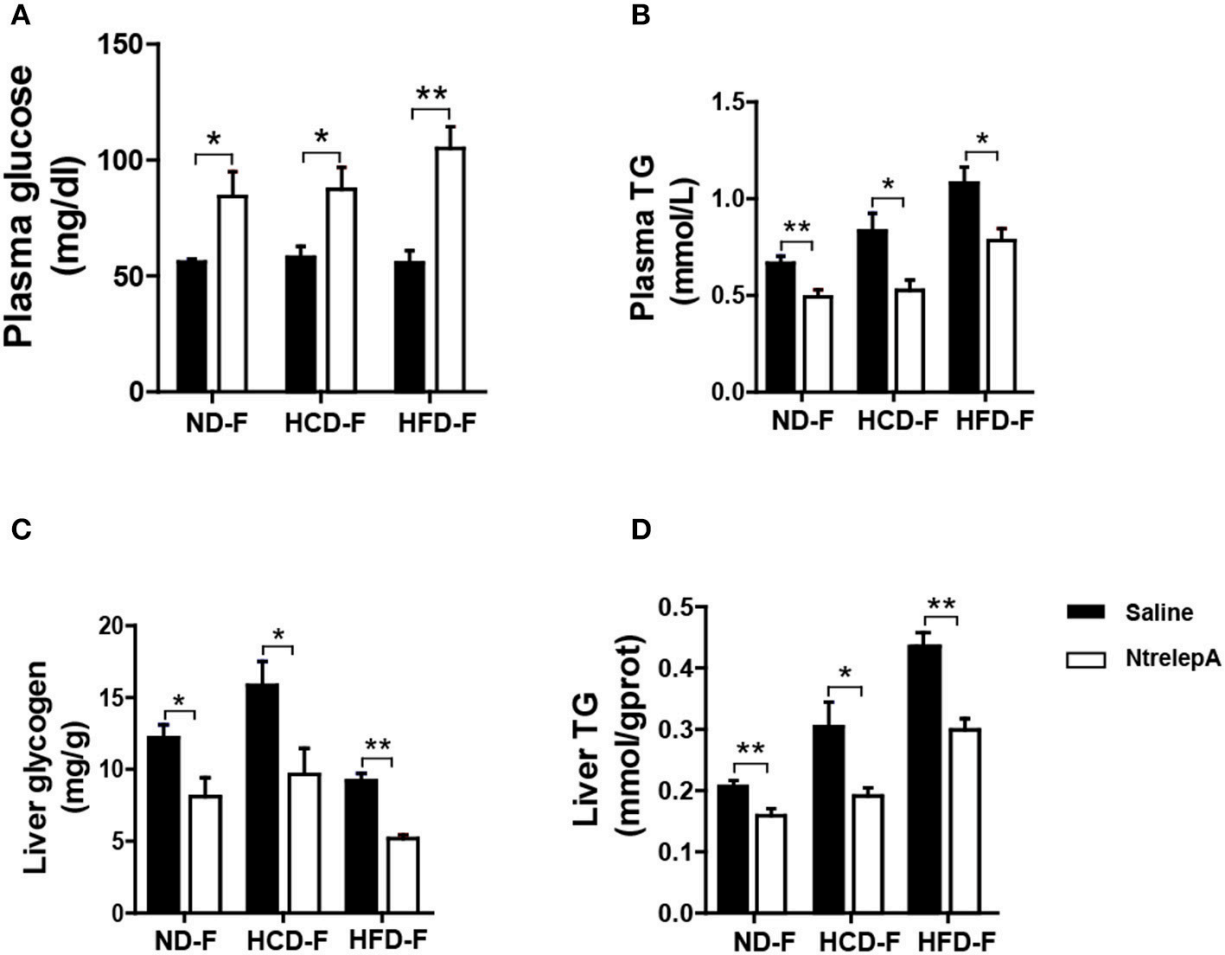
Recovered actions of injected NtrelepA in metabolism regulation after 1 week fasting in DIO Nile tilapia. **(A,B)** Plasma glucose and TG levels (*n* = 6). **(C,D)** Liver glycogen and TG content (*n* = 6). ^*^p < 0.05 and ^**^p < 0.01. Data shown as means ± SEM.

For animals in the wild, surplus calorie storage is essential to deal with unpredictable starvation. To determine whether the lost function of leptin in metabolic regulation could be recovered by fasting, fish were deprived of their diet for 1 week after 8 weeks of feeding, which were named as CD-F, HCD-F, and HFD-F groups. The fish were then i.p. injected with NtrelepA (400 ng/g BW) or saline to determine the action of leptin. Two hours after NtrelepA injection, the plasma glucose level increased significantly and the TG level decreased in all the three groups (Figures 8A,B). In response to this, liver glycogen and TG decreased significantly (Figures 8C,D). These results indicate that leptin could restore mobilization of the two major stored energy components to maintain normal physiological activities when the fish were deprived of feed.

## Discussion

In the present study, we confirmed that liver and SCAT are the two organs with higher expression of LepA in Nile tilapia. In DIO Nile tilapia, hyperleptinemia occurred along with the loss of the action of exogenous leptin to regulate food intake and body weight, demonstrating the existence of LR in Nile tilapia. More importantly, we found that HCD-DIO Nile tilapia retained the action of leptin in regulating lipid metabolism, while presenting LR in glucose metabolism regulation. Conversely, HFD-DIO Nile tilapia retained the action of leptin in regulating glucose metabolism while showed LR in lipid metabolism regulation. This indicates for the first time the selective metabolic regulation of leptin in DIO Nile tilapia with LR.

Leptin has been described as an adipostatic factor in mammals given its secretion predominantly by adipose tissue and in proportion to total adipose mass (21). However, the expression of leptin is barely detected in adipose tissue of chicken and quails (26). In addition, low level of leptin in liver and adipose tissue was also reported in frogs (27). In teleosts, liver has been considered to be the primary site for leptin synthesis, while there is almost no leptin expression in VAT (28). Here, using IHC analysis with a LepA-specific antibody, our data show that, besides liver, SCAT, a commonly underappreciated tissue with higher lipid catabolic activity than VAT (29) in Nile tilapia, also synthesized a high level of LepA. Remarkably, SCAT was also reported to contribute more leptin than visceral adipocytes in humans (30). In this regard, our findings established a connection between mammals and teleosts regarding the source of leptin.

In mammals, leptin regulates metabolic processes by reducing TG content and increasing FA β-oxidation (31), while increasing the incorporation of glucose into glycogen and reducing plasma glucose (32). However, the existing data in teleosts suggested that leptin stimulates glycogen depletion and elevates plasma glucose, but there are conflicting data on its lipolytic actions (10). Our data further confirmed that teleostean leptin induces not only glycolytic action, but also lipolytic action in teleosts. As endotherms, mammals have higher metabolic rates to maintain body temperature and normal physiological activities (33), and glucose is an efficient fuel for metabolism. Therefore, leptin in mammals may promote gluconeogenesis, but not glycogenolysis, which would allow glycogen stores to be conserved in the event of a sudden high energy demand (10). On the contrary, poikilotherms such as fish, cannot use glucose efficiently as a fuel (34) compared with mammals, which may explain the difference in the regulation of leptin in glycometabolism between teleosts and mammals. However, these findings merit further investigations to unveil the detailed mechanisms.

DIO, especially HFD-induced obesity with LR, has been discussed extensively in mammals (3). In the present study, Nile tilapia fed HCD or HFD for 8 weeks gained more body weight and body fat than ND-fed fish. In mammals, lipid accumulation can be promoted by periostin via inhibiting *PPAR*α gene transcription (35), while high level of *RBP4* also promotes lipid accumulation by inducing *PPAR*γ gene transcription (36). In our DIO Nile tilapia, HCD-fed fish had lower *PPAR*α and higher *periostin* expression, while HFD-fed fish had higher *PPAR*γ and *RBP4* expression. This shows that both HCD and HFD induced obesity, but the mechanisms may be different between the two models. Consistent with the findings in HFD-induced obese mice (37), in both DIO fish models hyperleptinemia appeared and exogenous leptin administration failed to regulate food intake and body weight. This confirms the existence of LR in Nile tilapia. Previous studies conducted on mammals defined LR as a loss of the ability of leptin to regulate food intake (3). However, studies also indicated that, in obesity with LR, leptin still retained actions that resulted in the development of hypertension, which is known as selective LR (38, 39). In addition, HFD fed mice reduced leptin-induced p-STAT3 in the arcuate nucleus, while retaining leptin sensitivity in other hypothalamic and extrahypothalamic nuclei (24). Moreover, a recent study demonstrated that DIO mice retained the ability of endogenous leptin to regulate food intake and body weight (5). Therefore, leptin may retain more of its activities in animals with LR than previously believed. It should be noted that the resistance to the metabolic actions of leptin has also been attributed to LR by default (39). Although studies demonstrated that the stimulatory effect of leptin on acetyl-CoA carboxylase activity or lipid oxidation was eliminated in HFD-induced DIO mice (40, 41), the action of leptin in glucose metabolism was not investigated in previous studies.

A key finding of the present study is the selective retention of the metabolic regulatory actions of leptin in two types of DIO Nile tilapia with LR. In the wild, animals have surplus calorie storage when food is abundant, which is critical to prolonging survival during times of famine (42). In other words, the rapid accumulation of body fat when food is abundant is an adaptive strategy of animals to deal with sudden food shortages. In C57BL/6J mice, HFD-induced obesity and LR are fully reversible once food intake is resumed on a chow diet, even with the same amount of calories intake as HFD group (43). This suggests that it is macronutrient composition that causes weight gain in DIO mice and not the total calories consumed (43). More recently, Riddle and colleagues reported that cavefish, Mexican tetra (*Astyanax mexicanus*) which carries a mutation in the insulin receptor lead to insulin resistance, and this phenotype is beneficial to weight gain for survival in nutrient-limited environment (44). Moreover, the occurrence of LR in zebrafish larvae promoted excessive caloric intake when food resources were plentiful, suggesting LR could be an ancient mechanism to promote somatic growth (45). In the present two different DIO Nile tilapia models, carbohydrate represents the main energy source in HCD while lipid is the main one in HFD. From an adaptive evolutionary perspective, hyperleptinemia may involve constant retention of the action to promote catabolism of the minor energy source while maintaining resistance to trigger catabolism of the primary energy source. This could provide a major benefit by helping animals to store the primary nutrient-containing energy and survive periods of food shortages. As an additional evidence, the selective metabolic LR disappeared when the HCD- or HFD-DIO Nile tilapia were fasted for 1 week, indicating that this selective metabolic action of leptin occurs when food is abundant. In mammals, the recovery of leptin sensitivity was in parallel with the recovery of NPY/AgRP and POMC neurons' response to leptin actions (43). In addition, fasted mice with low cellular SOCS-3 levels enhanced greatly leptin-induced p-STAT3 signals (46). Based on these findings, 1-week fasting might have improved the leptin-induced p-STAT3 signals and made the exogenous leptin recover its actions in metabolic regulation.

Overall, we conclude that, in HCD- and HFD-DIO Nile tilapia, hyperleptinemia and leptin resistance arise, but selective leptin activity in energy expenditure is retained. From an evolutionary and ecological perspectives, this finding of selective blockade to the metabolic action of leptin may reveal an adaptive strategy to store surplus calories efficiently.

## Author contributions

C-ZL and Z-YD designed research. C-ZL, L-JN, YL, and D-LL performed research. Z-YD, C-ZL, A-YH, M-LZ, and L-QC analyzed data. Z-YD and C-ZL wrote the paper.

### Conflict of interest statement

The authors declare that the research was conducted in the absence of any commercial or financial relationships that could be construed as a potential conflict of interest.

## Supplementary material

Figure S1 Identification of NtrelepA, preliminary experiment to determine the activity of NtrelepA. **(A)** Detection of the purified recombinant protein by SDS-PAGE. **(B)** Identification of the purified recombinant protein by MALDI-TOF-MS. **(C)** I.p. NtrelepA at dose of 200, 400, and 800 ng/g body weight on the effect of plasma glucose levels (*n* = 6) ***p* < 0.01. Data shown as means ± SEM.
