# Supplementary material for: Leptin Selectively Regulates Nutrients Metabolism in Nile Tilapia Fed on High Carbohydrate or High Fat Diet
Source: Front Endocrinol (Lausanne). 2018 Sep 27;9:574. doi: 10.3389/fendo.2018.00574 (PMC6201848; doi:10.3389/fendo.2018.00574)
Supplement: Supplementary file 1 [file Table_1.DOCX]

| **Table S1. The primers used in this study.** | | | | |
| --- | --- | --- | --- | --- |
| Usage | Gene name | Forward Primer (5' to 3') | Reverse Primer (5' to 3') | Size(bp) |
|  | *EF1α* | CTACGTGACCATCATTGATGCC | AACACCAGCAGCAACGATCA | 106 |
|  | *LepR* | ACGTGATGGAGGAGAGGGAGA | TCAGAGGGTGGATGGTGCA | 99 |
|  | *LepA* | ATGGCTGAACAGCTGGTGGTTA | GGAGGAAGTTCCATCCAAATCATC | 99 |
|  | *SOCS3* | ACCCTCAGTGTCAAGACAGCCTC | AGAACGCAGTCAAAGTGGGGAA | 121 |
|  | *PTP1B* | GGAATTGGGCGCTCTGGAA | CAGCACATCGCGAATACGAACT | 99 |
| qRT-PCR | *NPY* | GGACCTGGCCAAGTACTACTCAGC | ACAGCAGCTCTGAGACCAGTGTGT | 116 |
|  | *PPARα* | CTGATAAAGCTTCGGGCTTCCA | CGCTCACACTTATCATACTCCAGCT | 106 |
|  | *PPARγ* | TGGACTACACAAACATGCACAGC | CACGGGACTATCTGAGTACTGTGGA | 101 |
|  | *CPT1a* | TTTCCAGGCCTCCTTACCCA | TTGTACTGCTCATTGTCCAGCAGA | 102 |
|  | *apoE* | ATAAGCTGCAGAAGCGCCTCAATA | TTCACTGTATCCAGGTTCTGGGAG | 106 |
|  | *LPL* | CACCAAACTAGTGGGTCGTGATGT | TCCCAGACTATAACCCAGCAGATGA | 103 |
|  | *ATGL* | ACCAGCTTTCATGAGCTGCGTT | ACACATCTCAGCCAGCACCTTG | 117 |
|  | *Glut2* | CATTGGCATTCTAATCAGCCAGGT | TTGTAATATTGCTGGCGCTCCA | 106 |
| Plasmid | ^1^Forward Primer: GGAATTCCATATGCACCACCACCACCACCACGCTCCTTTGCCAGTGGAAGTA | | | |
| construction | ^2^Reverse Primer: CCGCTCGAGTCAGCAAGTCTCCAGCTGATCCA | | | |

^1^ NdeI restriction-enzyme site; ^2^XhoI restriction-enzyme site.
