# Supplementary material for: Leptin Selectively Regulates Nutrients Metabolism in Nile Tilapia Fed on High Carbohydrate or High Fat Diet
Source: Front Endocrinol (Lausanne). 2018 Sep 27;9:574. doi: 10.3389/fendo.2018.00574 (PMC6201848; doi:10.3389/fendo.2018.00574)
Supplement: Supplementary file 2 [file Table_2.DOCX]

| **Table S2. The formulation of the experimental diets.** | | | |
| --- | --- | --- | --- |
| Component(g/kg) | ND | HCD | HFD |
| Casein | 229 | 229 | 229 |
| Gelatin | 58 | 58 | 58 |
| Fish meal | 120 | 120 | 120 |
| Soybean oil | 15.2 | 15.2 | 75.2 |
| Fish oil | 15.2 | 15.2 | 75.2 |
| Corn starch | 329.15 | 449.15 | 329.15 |
| Vitamin premix^1^ | 15 | 15 | 15 |
| Mineral premix^2^ | 28.2 | 28.2 | 28.2 |
| CMC | 25 | 25 | 25 |
| Cellulose | 160 | 40 | 40 |
| Choline chloride | 5 | 5 | 5 |
| BHT | 0.25 | 0.25 | 0.25 |
| Total | 1000 | 1000 | 1000 |
| Composition |  |  |  |
| Dry matter (%) | 93.22 | 92.81 | 93.16 |
| Crude protein (%) | 36.51 | 36.84 | 36.58 |
| Crude lipid (%) | 4.12 | 4.51 | 16.29 |
| Ash (%) | 5.18 | 5.36 | 5.31 |

^1^ Mineral premix, (g/kg): 314.0 g CaCO_3_; 469.3 KH_2_PO_4_; 147.4 g MgSO_4_·7H_2_O; 49.8 g NaCl; 10.9 g Fe(II) gluconate; 3.12 g MnSO_4_·H_2_O; 4.67 g ZnSO_4_·7H_2_O; 0.62 g CuSO_4_·5H_2_O; 0.16 g KJ; 0.08 g CoCl_2_·6H_2_O; 0.06 g NH_4_ molybdate; 0.02 g NaSeO_3_.

^2^ Vitamin premix, (mg or IU/kg): 500,000 I.U. (international units) Vitamin A, 50,000 I.U. Vitamin D3, 2500 mg Vitamin E, 1000 mg Vitamin K3, 5000 mg Vitamin B1, 5000 mg Vitamin B2, 5000 mg Vitamin B6, 5000 μg Vitamin B12, 25,000 mg Inositol, 10,000 mg Pantothenic acid, 100,000 mg Cholin, 25,000 mg Niacin, 1000 mg Folic acid, 250 mg Biotin, 10,000 mg Vitamin C.
