# Supplementary figures and images for: Leptin Selectively Regulates Nutrients Metabolism in Nile Tilapia Fed on High Carbohydrate or High Fat Diet
Source: Front Endocrinol (Lausanne). 2018 Sep 27;9:574. doi: 10.3389/fendo.2018.00574 (PMC6201848; doi:10.3389/fendo.2018.00574)

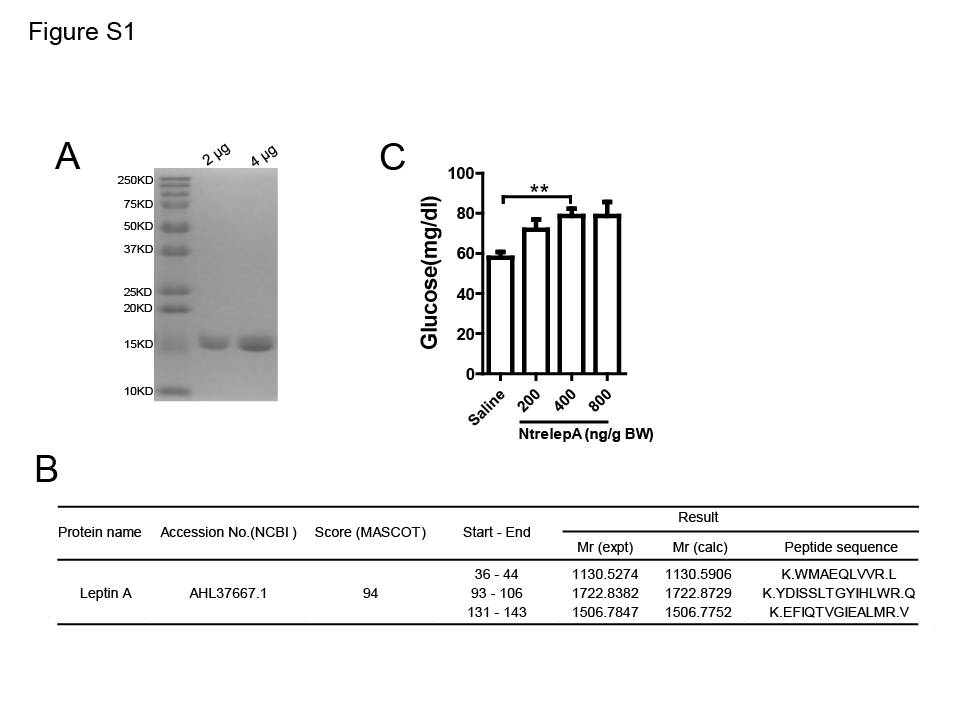

Supplement: Figure S1 — Identification of NtrelepA, preliminary experiment to determine the activity of NtrelepA. (A) Detection of the purified recombinant protein by SDS-PAGE. (B) Identification of the purified recombinant protein by MALDI-TOF-MS. (C) I.p. NtrelepA at dose of 200, 400, and 800 ng/g body weight on the effect of plasma glucose levels (n = 6) **p < 0.01. Data shown as means ± SEM. [file Image_1.TIF]
